# Artificial Ageing Methodologies for Zirconia-Based Restorations in Fixed Prosthodontics: A Narrative Review and Proposal of the MIAZE Reporting Framework

**DOI:** 10.3390/jfb17100515

**Published:** 2026-10-13

**Authors:** Marta Banjšak, Mark Žic, Marko Jakovac

**Affiliations:** 1 Department of Fixed Prosthodontics, School of Dental Medicine, University of Zagreb, Gundulićeva 5, 10000 Zagreb, Croatia; mbanjsak@sfzg.hr; 2 Ruđer Bošković Institute, Bijenička Cesta 54, 10000 Zagreb, Croatia

**Keywords:** zirconia, fixed prosthodontics, artificial ageing, hydrothermal degradation, thermocycling, chewing simulation, low-temperature degradation, phase transformation, in vitro testing

## Abstract

Zirconia-based ceramics are widely used in fixed prosthodontic restorations owing to their favourable mechanical properties and evolving aesthetic characteristics. However, predicting long-term performance remains challenging because artificial ageing methodologies are highly heterogeneous and existing standards do not fully reflect the diversity of modern zirconia systems, including high-translucency and multilayer materials; variations in experimental conditions, specimen designs, measurement approaches, and reporting practices limit inter-study comparison and quantitative evidence synthesis. This review examines current artificial ageing methodologies for zirconia-based restorations and identifies a fundamental limitation beyond the absence of a universal ageing protocol: the lack of a standardised framework for preserving and exchanging experimental information. To address this, the Minimum Information About a Zirconia Experiment (MIAZE) reporting framework is proposed as a model-neutral, machine-readable structure for documenting experimental observations and associated metadata. MIAZE is proposed as a minimal reporting framework for prospective use that does not require identical experimental protocols, instrumentation, or analytical approaches, but defines the essential information needed to preserve scientific interpretability and enable future dataset reuse. By separating data documentation from subsequent interpretation, MIAZE is designed to provide a foundation for reproducibility, independent verification, quantitative meta-analysis, and the application of diverse analytical approaches, shifting the focus from a single universal ageing protocol toward interoperable experimental datasets capable of supporting evolving zirconia materials, methodologies, and computational tools. Literature was identified through PubMed searches (2000 to 2026) combining zirconia, dental, and ageing terms, supplemented by reference screening; the review is narrative and not exhaustive. MIAZE is presented as a draft proposal for prospective reporting; its benefits have not yet been demonstrated on multi-laboratory datasets, and an illustrative assessment of published studies shows that legacy data can be encoded only incompletely, which motivates prospective use.

## 1. Introduction

Yttria-stabilised tetragonal zirconia polycrystal (Y-TZP) was introduced into dentistry in the early 2000s and has since become the material of choice for a wide range of fixed prosthodontic restorations, from single crowns to multi-unit fixed dental prostheses (FDPs) [1,2]. Its clinical success is attributable to exceptional flexural strength, fracture toughness via the tetragonal-to-monoclinic (t → m) transformation-toughening mechanism, biocompatibility, and a continuously improving aesthetic potential enabled by successive material generations [3,4]. Long-term clinical follow-up supports the use of tooth-supported zirconia restorations across single crowns and multi-unit FDPs [5,6].

A fundamental concern with all Y-TZP formulations is their susceptibility to hydrothermal degradation, also termed low-temperature degradation (LTD) or ageing. LTD refers to the slow, water-assisted t → m transformation that nucleates at the surface of metastable tetragonal grains and propagates inward at temperatures close to body temperature; autoclave ageing is used to accelerate this process in the laboratory. In the oral environment, zirconia restorations are continuously exposed to moisture, thermal fluctuations, masticatory forces, and chemical challenges from food, beverages, and saliva. These stressors can drive a spontaneous, surface-initiated t → m phase transformation, accompanied by an approximately 4% to 5% volume expansion that generates compressive stresses and microcracking at the surface, progressively roughening the material and diminishing mechanical properties in susceptible compositions [7,8].

In vitro artificial ageing is designed to simulate years of in vivo service within a manageable experimental timeframe. However, zirconia in the specific context of fixed prosthodontics lacks a dedicated, universally accepted ageing protocol. The existing ISO 13356 standard [9], originally developed for orthopaedic implants, addresses the characterisation of Y-TZP for biomedical applications and specifies hydrothermal ageing at 134 °C and 2 bar for 5 h. While this provides a valuable comparative benchmark, its applicability to the complex prosthetic loading environment of the oral cavity is limited: ISO 13356 evaluates a single, fixed hydrothermal condition originally calibrated for orthopaedic-grade 3Y-TZP, and it does not fully account either for the distinct ageing behaviour of contemporary translucent, high-yttria zirconia generations or for the combined thermal, chemical, and mechanical stressors present intraorally [10,11].

This is a narrow, protocol-level limitation, but it is symptomatic of a broader, field-wide problem. The increasing diversity of zirconia materials and artificial ageing methodologies suggests that the principal challenge is not merely the absence of a universal testing protocol, but the inability to consistently preserve, compare, and reuse experimental information generated under heterogeneous conditions. Existing ISO test standards include test-report requirements, but these are specific to each test method and are not designed to support aggregation of results across studies or machine-readable reuse. This is the gap that the reporting framework introduced below addresses. Formal consensus standards are, moreover, structurally unable to keep pace with the rate at which new zirconia generations and commercial formulations reach the market. Compounding this, commercial dental zirconia is produced under proprietary, undisclosed manufacturing parameters; because dopant profiles and sintering technologies are not shared between companies, each product functions as a compositional “black box”, and independent characterisation must rely on empirical benchmarking of the finished material rather than reverse-engineering its formulation (see Section 2.1).

This methodological gap has led to a proliferation of heterogeneous ageing protocols throughout the dental literature. Because these protocols vary in temperature, pressure, duration, thermocycle number, mechanical loading parameters, chemical agents, specimen geometries, and their combinations (a single study may, for example, apply autoclave ageing alongside a sequential protocol of chewing simulation, thermocycling, and acid immersion [12]), direct inter-study comparisons are extremely challenging [11,13,14,15,16]. This reinforces the need for the standardised reporting layer proposed in this work (Section 4).

Accordingly, this narrative review analyses the principal artificial ageing methodologies applied to zirconia-based materials in fixed prosthodontics, evaluates their mechanistic rationale and clinical relevance, and identifies the major sources of methodological heterogeneity. Rather than proposing another universal ageing protocol or a replacement for existing ISO standards, this work proposes a structured, machine-readable reporting framework, the Minimum Information About a Zirconia Experiment (MIAZE), in which experimental observations and metadata are preserved in an open JavaScript Object Notation (JSON) format, independent of the specific protocol used to generate them. By establishing this standardised data infrastructure, experimental datasets generated across different laboratories can be preserved, shared, compared, and independently reanalysed, remaining flexible enough to accommodate the continuous evolution of both experimental protocols and zirconia materials themselves.

This narrative review is based on PubMed searches (January 2000 to September 2026) using combinations of zirconia/Y-TZP, dental/prosthodontic, and ageing terms (hydrothermal, low-temperature degradation, thermocycling, chewing simulation, chemical immersion, UV), supplemented by backward citation screening. In vitro and clinical studies on zirconia for fixed prostheses were considered; zirconia implants and orthopaedic applications were included only where they inform test standards. The search strings used for the bibliometric analysis in Section 2.2 are provided in Supplementary Section S5.

## 2. Material Context and Current Methodologies

### 2.1. Zirconia Generations and the Commercial “Black Box”

Understanding artificial ageing requires evaluating how zirconia composition and microstructure influence susceptibility to low-temperature degradation. This section summarises the four compositional zirconia classes commonly used in prosthodontics [17,18,19]: conventional 3Y-TZP (first generation), translucent 3Y-TZP (second generation), 5Y-TZP (third generation), and 4Y-TZP (fourth generation), numbered by clinical introduction rather than by yttria content. Increasing yttria content promotes stabilisation of the cubic phase, enhancing translucency and improving resistance to LTD at the expense of transformation toughening and, consequently, flexural strength [17,20,21]. Structural design (monolithic vs. multilayer) is independent of compositional class: any formulation may be manufactured as either.

**Figure 1:**
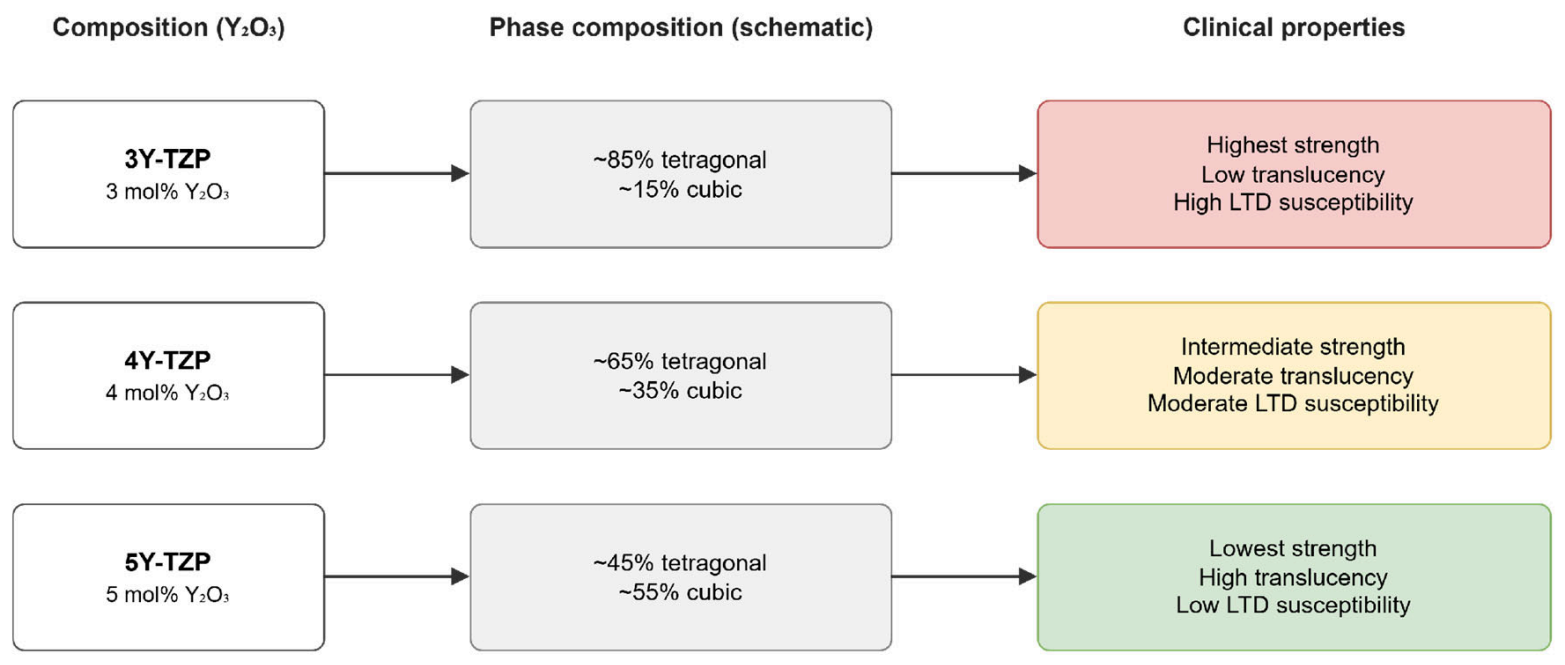
Schematic phase composition and clinical properties of dental zirconia as a function of yttria content [17,20,21]. Phase fractions are indicative and vary with processing conditions and manufacturer.

Figure 1 shows this stabilisation relationship directly: as yttria content rises from 3 to 5 mol%, the cubic phase fraction increases at the expense of the transformable tetragonal phase that drives both toughening and LTD susceptibility. This compositional shift forms the primary axis along which the four generations described below are positioned.

3Y-TZP (First and Second Generation). The original first-generation formulation (3 mol% Y_2_O_3_, ~0.25 wt% Al_2_O_3_) has the highest proportion of transformable tetragonal phase and high LTD susceptibility, historically used as a veneered coping material rather than monolithically [17,20]. Second-generation 3Y-TZP increased translucency by reducing Al_2_O_3_ content (~0.05 wt%) and raising sintering temperature, which increases grain size and consequently heightens LTD susceptibility relative to first-generation compositions [22]. Surface treatment (e.g., sandblasting) prior to ageing further alters morphology and residual stress, influencing subsequent degradation kinetics [23,24].

5Y-TZP and 4Y-TZP (Third and Fourth Generation). 5Y-TZP (5 mol% yttria) stabilises more than 50% of the microstructure in the cubic phase, dramatically increasing translucency while diminishing the transformation-toughening mechanism (flexural strength 400 to 900 MPa), but yielding substantially greater resistance to hydrothermal ageing [17]. Standard autoclave ageing produces little or no t → m conversion in most 5Y-TZP materials [21,25,26]. However, the tetragonal grains remaining in 5Y-TZP are yttria-lean and remain transformable, and a gradual increase in monoclinic content with autoclave ageing time (up to 10 h) has been reported for colour-gradient multilayer blocks, including a 5Y-TZP product [27]; LTD should therefore be regarded as reduced, not absent, in this generation. Optical properties remain similarly stable after prolonged ageing [13,28,29,30]. 4Y-TZP (4 mol% yttria) was developed as a compromise, offering intermediate translucency, mechanical performance, and hydrothermal ageing resistance between 3Y-TZP and 5Y-TZP [31], allowing clinicians to balance aesthetics with durability where high-strength requirements preclude 5Y-TZP.

Multilayer Zirconia. Contemporary strength-gradient multilayer systems typically combine a 3Y- or 4Y-TZP cervical and body region with a 4Y- or 5Y-TZP incisal or occlusal region to optimise strength and aesthetics simultaneously; colour-gradient systems, by contrast, vary pigmentation within a single composition. Ageing behaviour is layer-dependent: 3Y-TZP and transitional layers undergo meaningful t → m transformation upon hydrothermal ageing, while 4Y-TZP/5Y-TZP layers remain largely resistant, even after extended autoclave exposure [32,33,34]. This heterogeneous, layer-dependent response within a single restoration has direct implications for how multilayer systems should be aged and characterised in laboratory studies [35,36,37].

**Figure 2:**
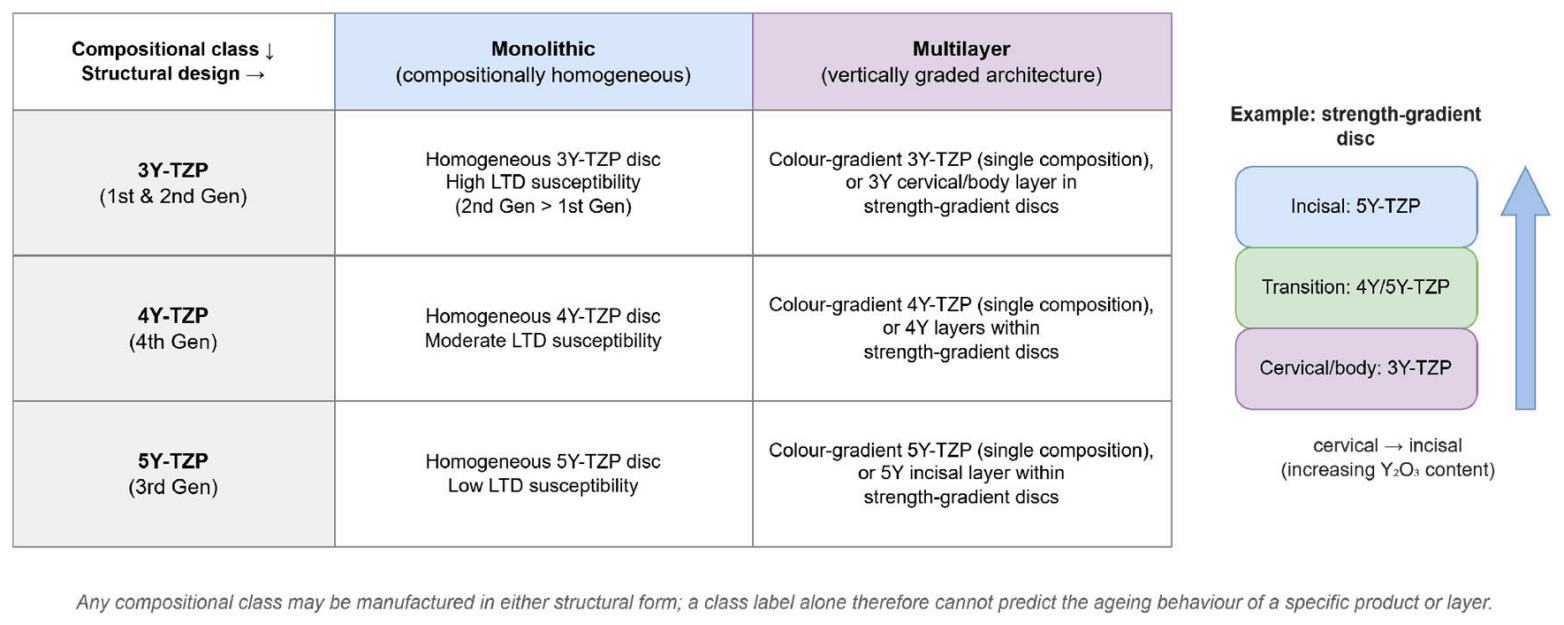
Classification of dental zirconia along two independent axes: (i) compositional class, defined by yttria content, and (ii) structural design, that is, monolithic (compositionally homogeneous) versus multilayer (vertically graded architecture). Any compositional class may be manufactured in either structural form, so a single generation label alone cannot predict a restoration’s ageing behaviour. An example strength-gradient disc illustrates the cervical-to-incisal increase in yttria content.

Figure 2 makes explicit that these two axes are independent: any of the compositional classes above may be manufactured as either a homogeneous monolithic disc or a graded multilayer disc, which is precisely why a single generation label is not sufficient to predict a restoration’s ageing behaviour once structural design is also in play.

Implication for benchmarking. Because manufacturing parameters, dopant profiles, and sintering technologies are not disclosed by manufacturers, commercial zirconia discs behave as compositional black boxes despite belonging to a known generation class. Materials nominally in the same generation can therefore exhibit materially different ageing susceptibility depending on undisclosed processing variables. This problem compounds further in multilayer systems, where a single disc contains several compositionally distinct, independently ageing zones (see Multilayer Zirconia, above): black-box opacity at the product level and layer-level compositional heterogeneity together mean that benchmarking cannot treat a restoration as a single material. What is required is a reporting approach flexible enough to document each layer or material zone as its own discrete experimental unit, rather than collapsing a multilayer specimen into one whole-restoration data point. This reinforces that meaningful comparison across commercial products must rely on standardised empirical benchmarking of finished materials, at the resolution of the individual layer where relevant, which is only possible if the resulting data are reported in a consistent, reusable format (see Section 4). In practice, the black-box limitation can be partly mitigated by characterising each as-received, as-sintered material before ageing. Feasible routine steps include X-ray diffraction with Rietveld refinement to quantify the initial tetragonal, cubic, and monoclinic fractions and the tetragonal lattice parameters; scanning electron microscopy of polished, thermally etched surfaces to determine mean grain size (e.g., linear-intercept method); Archimedes density; and, where available, X-ray fluorescence or energy-dispersive spectroscopy to verify yttria and alumina content. Together with the lot number and the sintering programme actually used, these data form a minimal material fingerprint that is recorded in the MIAZE material_baseline block (Section 4). Because processing history can change microstructure and phase content even for nominally identical compositions, MIAZE records the sintering programme actually applied (peak temperature, dwell, heating and cooling rates) and, where available, the tetragonal lattice parameters, which reflect yttria partitioning between tetragonal and cubic grains and thus transformability [21].

### 2.2. Survey of Ageing Protocols and Data-Representation Gaps

**Figure 3:**
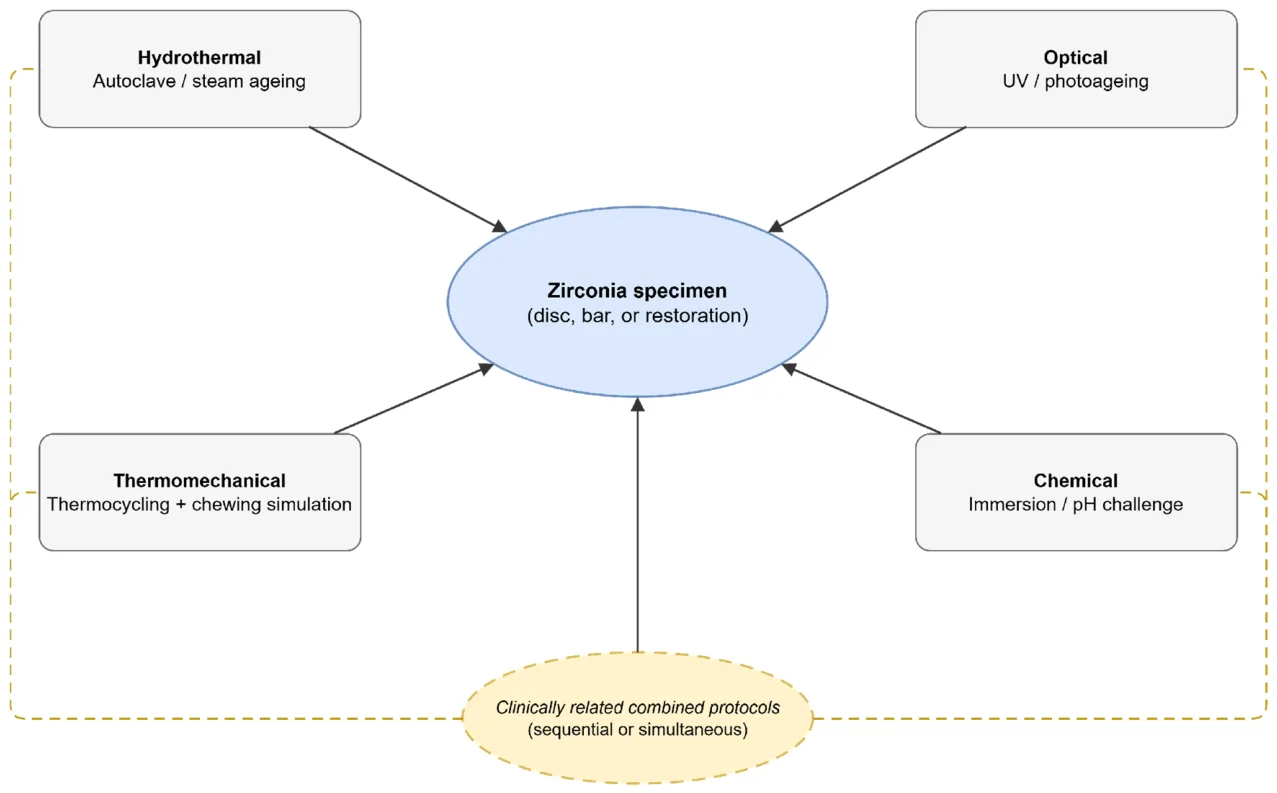
Principal artificial ageing domains applied to dental zirconia. Each of the four domains (hydrothermal; thermomechanical, comprising thermocycling and chewing simulation; chemical; and optical) acts on the zirconia specimen (disc, bar, or restoration). Dashed lines indicate that clinically related combined protocols draw on two or more domains, sequentially or simultaneously. Corresponding test standards are listed in Table 1.

Artificial ageing methods for dental zirconia can be grouped into four stress domains (hydrothermal; thermomechanical, comprising thermocycling and chewing simulation; chemical; and optical), complemented by combined protocols that act on more than one domain (Table 1, Figure 3).

Figure 3 maps these four domains schematically around a central specimen, with dashed lines marking the domains on which combined, multi-stage protocols (discussed below) draw; the individual methods are detailed in turn below.

Hydrothermal Ageing (Autoclave). The principal method for evaluating LTD, exposing specimens to saturated steam to induce surface-to-bulk t → m transformation. While ISO 13356:2015 specifies benchmark conditions (134 °C, 2 bar, 5 h), reported parameters vary considerably (110 to 140 °C; 1 to 64 h), and because time–temperature conversion factors were calibrated on 3Y-TZP, they cannot be transferred to 4Y- and 5Y-TZP, for which a fixed 5 h exposure may under-challenge the material [43,44]. ISO 13356:2015 specifies that, after this exposure, the monoclinic content must not exceed 25% and strength must not decrease by more than 20%, with minimum post-ageing values of 500 MPa in biaxial flexure or 800 MPa in four-point bending.

Thermocycling. Simulates dietary temperature fluctuations (typically 5 to 55 °C) to assess thermal fatigue, primarily at bonded interfaces since bulk zirconia shows minimal structural change. Protocols in the dental literature are unstandardised, with cycle numbers ranging from 600 to 50,000 [14,45,46,47].

Mechanical Ageing (Chewing Simulation). Replicates cyclic fatigue and moisture-assisted subcritical crack growth. Common protocols use 1.2 million cycles (≈5 years simulated function) at 50 to 200 N, but significant variability exists in load magnitude, antagonist material, and layer-specific yttria content across studies [48,49,50,51]. No ISO standard specifies chewing simulation of tooth-supported zirconia restorations. ISO 14801 is sometimes cited for loading parameters, but it describes dynamic fatigue testing of endosseous dental implants under a defined off-axis load and does not address antagonist contact, sliding, or crown and FDP geometry; it therefore offers only general guidance on cyclic loading.

Chemical Immersion. Chemical immersion (e.g., 4% acetic acid, HCl) simulates acidic challenge via kinetic mechanisms distinct from hydrothermal ageing [52].

Photoageing. UV photoageing primarily assesses extrinsic colour/cement stability rather than intrinsic structural longevity [53,54]. Combined thermomechanical or multi-stage protocols introduce further methodological variables.

The extent of LTD also depends on how it is measured. Conventional Bragg–Brentano XRD samples an information depth of several micrometres and yields an averaged monoclinic fraction, whereas grazing-incidence XRD provides depth profiles, Raman micro-spectroscopy offers micrometre-scale spatial mapping, and cross-sectional SEM or focused ion beam (FIB) imaging measures transformed-layer thickness directly. Results also vary with the ageing medium (water, artificial saliva), surface finish, shading, and speed-sintering protocols. This review does not systematically cover shaded and speed-sintered materials or zirconia implants, and most cited studies used discs or bars rather than restoration-shaped specimens, which limits direct extrapolation to crowns and FDPs.

**Figure 4:**
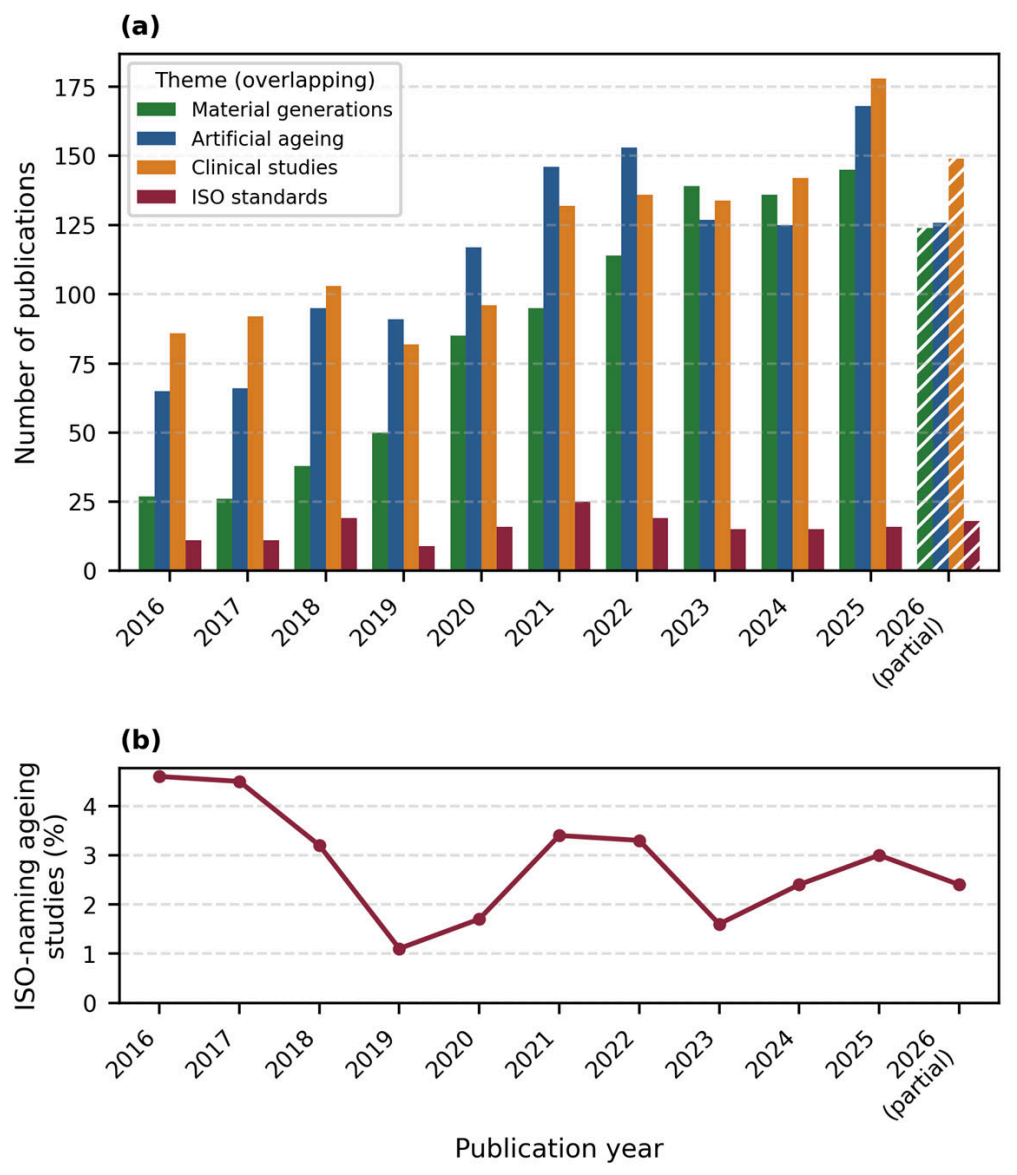
Bibliometric trends in dental zirconia research indexed in PubMed, 2016 to 2026. (**a**) Annual number of publications per theme, identified by title/abstract searches; themes overlap, so a publication can be counted in more than one theme. (**b**) Percentage of artificial-ageing studies naming an ISO standard in the title or abstract; annual counts were small (1 to 5 studies), so year-to-year variation should not be over-interpreted. Values for 2026 (hatched) are partial. Search strings and annual counts are provided in Supplementary Section S5; data retrieved on 23 September 2026.

The Fragmentation Gap. A PubMed analysis of title and abstract records from 2016 to 2025 (search strategy in Supplementary Section S5) shows that studies on artificial ageing of dental zirconia increased 2.6-fold, from 65 to 168 per year (Figure 4a), whereas only 32 of 1153 ageing studies (2.8%) named an ISO standard in the title or abstract, with no upward trend (1.1% to 4.6% per year; Figure 4b). Because studies may follow a standard without naming it in the abstract, and because PubMed covers materials-science journals incompletely, these counts indicate trends rather than a complete census. Beyond publication volume, the reusability of this literature is limited by what it reports. In the five studies examined in Supplementary Material (Table S1), four of which were our own, measurement uncertainty was not reported in any study, the disc lot number was stated in one, and per-specimen values were absent from both studies with destructive endpoints; because only one of these studies involved hydrothermal ageing, this small sample illustrates the problem but does not establish its prevalence. Recovering such information requires manual reading of unstructured text, which is slow and prone to inconsistent interpretation [55]; where a parameter was never reported, it cannot be recovered at all.

The heterogeneity is not confined to protocol selection; it extends through every stage of experimental design and outcome reporting:

Time–temperature equivalency remains contested: the widely used conversion estimate of 1 h at 134 °C ≈ 3 to 4 years in vivo [8] conflicts with long-term ageing data obtained at body temperature, which show linear, continuous degradation at 37 °C without retardation and indicate that the commonly quoted equivalence between steam sterilisation at 134 °C and decades of in vivo service cannot be verified from X-ray diffraction data [56]. This uncertainty materially changes the clinical interpretation of identical experiments. Reported autoclave conditions also vary widely between studies [11,16], and apparatus-level variables such as chamber volume, pressure regulation, heating ramp rate, and water volume are not specified in ISO 13356, so they may differ between laboratories even when the nominal temperature, pressure, and duration are identical.

Generation-specific susceptibility means a single hydrothermal protocol cannot meaningfully stress both 3Y-TZP and 5Y-TZP: standard 5 h ageing effectively challenges 3Y-TZP but barely perturbs 5Y-TZP, risking the false impression that high-translucency zirconia is inherently indestructible. Multilayer systems compound this, as a single whole-restoration protocol cannot represent the appropriate stress for compositionally distinct layers within the same disc [32,33].

Thermocycling parameters span 600 to 50,000 cycles, dwell times of 3 to 60 s, and temperature ranges from 5 to 55 °C up to 5 to 75 °C, a heterogeneity explicitly recognised in a landmark review as rendering inter-study comparison essentially impossible [15].

Chewing simulation variables, including load magnitude, frequency, antagonist material, and loading geometry, vary substantially, and the reference convention of 1.2 × 10^6^ cycles representing approximately 5 years is itself only an approximation, because it rests on an assumed annual number of masticatory cycles [50].

Outcome reporting is as heterogeneous as protocol selection: phase transformation is quantified via different XRD methods with differing reference intensities; flexural strength testing employs three-point, four-point, and biaxial configurations with different span lengths and loading rates; colour assessment uses different illuminants, apertures, and formulae (ΔE CIE 1976 vs. ΔE_00_ CIE 2000).

Because of this compounding heterogeneity, spanning protocol selection, environmental control, and outcome quantification simultaneously, a quantitative synthesis across the full range of protocols, materials, and outcomes covered by this review was not feasible, although meta-analyses restricted to specific outcomes and material classes have been published [13,16]. This is not merely an inconvenience for narrative synthesis: it is the central symptom that motivates the diagnosis and proposal developed in the remainder of this work.

## 3. Core Gaps and Root-Cause Analysis

A persistent limitation across the literature is the absence of a dedicated standard specifically addressing the artificial ageing of zirconia in fixed prosthodontics. Existing standards, such as ISO 13356, were originally developed for general biomedical applications and fail to capture the mechanical loading conditions, compositional complexity, or clinical heterogeneity of modern dental systems, particularly multilayer and high-translucency materials. This regulatory vacuum has produced a landscape dominated by proprietary, non-standardised protocols: because dental research currently operates in isolated silos, experimental data are frequently trapped within unique, study-specific formats, typically free-text descriptions and static, non-machine-readable summary tables, often reporting only condensed outcomes rather than the underlying material baseline, experimental increments, or response observations needed for independent reuse. This is a distinct problem from the manufacturer-level opacity discussed in Section 2.1: there, the barrier is undisclosed composition; here, the barrier is how researchers who do have full access to their own raw data choose to report it.

The same disconnect reappears one level up, between the laboratory and the clinic. Validating in vitro ageing protocols requires correlating laboratory-derived kinetic data with the documented service history of clinically retrieved zirconia restorations, yet this correlation is only as good as the resolution at which the original laboratory data were captured. Where studies report only pass/fail outcomes or condensed summary statistics, there is no way to retrospectively align them with retrieval findings, regardless of how good the underlying experiment was. This is not a shortage of ageing studies; it is a shortage of ageing data preserved at a resolution fine enough to be reused for a question the original authors did not anticipate.

Table 2 illustrates what this correlation could look like in practice, once laboratory data are captured at sufficient resolution, but the matrix itself presupposes the very reporting granularity that current summary-outcome-only publications do not provide.

Both symptoms, namely the absence of a fixed-prosthodontic-specific standard and the lab-to-clinic disconnect, trace back to a single conflation. In practice, reporting and interpretation are frequently merged: studies commonly express hydrothermal exposure as “equivalent clinical years” derived from a single conversion factor, typically 1 h at 134 °C ≈ 3 to 4 years in vivo [25,27], or report compliance with a threshold rather than the measured values, so that the interpretation cannot be separated from the data. But no protocol, however carefully designed, can be adopted fast enough: formal ISO consensus mechanisms operate on a timescale of years, while commercial zirconia generations and formulations turn over continuously. Chasing protocol consensus is therefore chasing a moving target. The true barrier to comparable, reusable, clinically correlatable zirconia data is not the absence of the right analytical method; it is structural: the absence of a common format in which any method’s inputs and outputs can be recorded, regardless of which protocol produced them. It is this specific structural void, not a shortage of candidate testing protocols, that the MIAZE framework proposed below is designed to fill.

Table 3 makes this conflation concrete: for each recurring methodological limitation in the ageing literature (speculative clinical time extrapolation, equipment-specific variability, heterogeneous specimen geometries, and qualitative or pass/fail reporting), a corresponding analytical approach already exists in principle, but remains inapplicable in practice because the underlying raw observations were never captured in a form that supports it. Section 5.1 returns to this table to show what becomes possible once that capture problem is solved.

This reframing is the basis for the MIAZE framework, proposed here as a foundational data infrastructure layer rather than a competing testing protocol. MIAZE is not designed to resolve which ageing protocol, time–temperature conversion factor, or outcome metric is “correct”; it is designed so that whichever choices a given study makes, the resulting observations are captured in a form that remains interoperable with every other study’s choices. By prioritising semantic interoperability over protocol uniformity, MIAZE allows studies that share an objective but differ in experimental design to still be mapped against one another.

**Figure 5:**
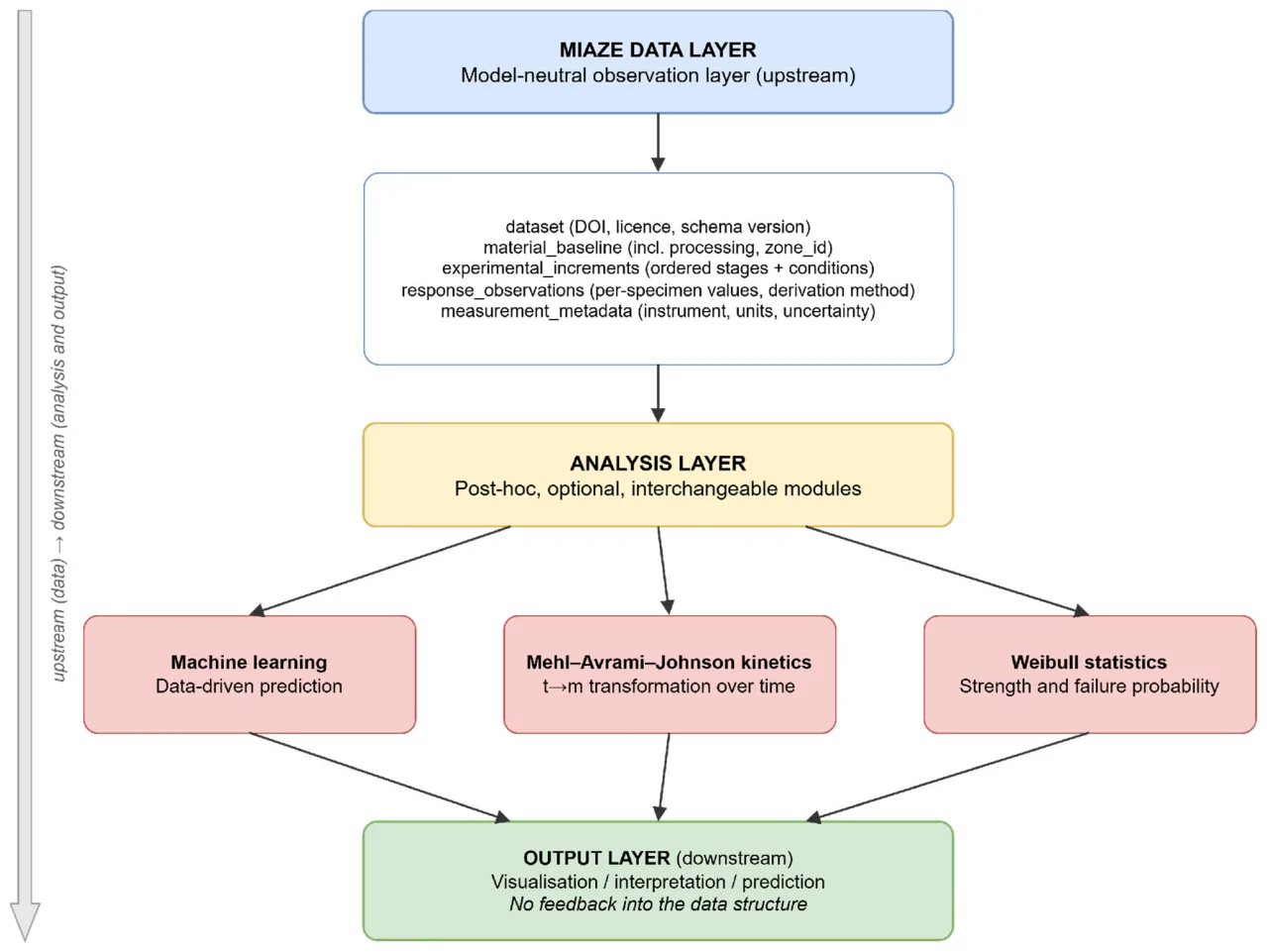
Layered architecture of zirconia experimental data reporting and analysis. The MIAZE data layer (upstream) records the dataset, material baseline, experimental increments, response observations, and measurement metadata. Post hoc analytical modules, shown here as machine learning, Mehl–Avrami–Johnson transformation kinetics [57], and Weibull statistics [58], operate downstream as optional, interchangeable modules; their outputs do not feed back into the data structure.

Critically, MIAZE functions as a structural prerequisite for interoperability, not an analytical method in itself. It embeds no assumptions about degradation mechanisms, kinetic models, or statistical approach. Instead, it defines a clean, machine-readable dataset boundary below which raw observations are recorded, and above which any analytical toolset, such as kinetic modelling, statistical regression, machine learning, or direct clinical retrieval correlation, can operate independently, without being constrained or presupposed by the reporting layer itself (Figure 5). This separation is what makes the framework proposed in the remainder of this work fundamentally different from a new ageing protocol: it does not ask the field to agree on how to test zirconia, only on how to record what was done and what was observed.

## 4. The MIAZE Framework

MIAZE is a proposed minimal, model-neutral reporting framework for zirconia-based experimental studies in fixed prosthodontics. Its purpose is to preserve the scientific interpretability, reproducibility, and future reusability of experimental datasets, regardless of which ageing protocol, instrumentation, or analytical method produced them. Critically, MIAZE does not require researchers to adopt identical experimental protocols or analytical approaches; it provides a standardised structure for documenting what was done and what was observed, allowing heterogeneous studies to remain comparable while preserving the flexibility each research group needs for its own objectives.

MIAZE specifies data reporting only. It does not prescribe analytical frameworks, mathematical models, or interpretation strategies; these remain independent and may be selected according to the dataset and the scientific question at hand.

This separation is enforced by five core reporting principles:

1. Observation primacy: measured quantities, including conventionally derived values, are reported together with their derivation method, whereas model-fitted parameters are excluded.
2. Model neutrality: the structure is independent of any specific kinetic, statistical, or machine-learning model.
3. Interoperability: data are encoded in a structured key–value format (JSON or equivalent) using controlled vocabularies, Unified Code for Units of Measure (UCUM) codes, and numeric uncertainty objects.
4. Complete metadata context: experimental conditions, instrumentation, specimen provenance, and processing history are fully documented.
5. Explicit non-reporting: unreported values are recorded as null, so that missing information remains visible.

Together, these principles align MIAZE with the broader FAIR data principles [59] (findable, accessible, interoperable, reusable), extending zirconia reporting beyond static publication toward long-term, computationally accessible datasets.

MIAZE is a prospective proposal: it defines how future studies should record their data and is not intended as a tool for re-encoding the existing literature. Its role is comparable to that of a JSON Schema, which specifies the structure of documents created against it; documents written before the schema existed can often be read, but rarely conform to it. Published zirconia ageing studies behave in the same way. They can be mapped onto the MIAZE structure, but in the five studies examined in Supplementary Section S4, essential parameters such as measurement uncertainty, per-specimen values, and disc lot numbers were mostly not reported and could not be recovered; how common such omissions are across the wider literature has not been quantified. This under-reporting is the main reason MIAZE is needed: until now, no common reporting structure existed for zirconia ageing studies, and the missing information can be preserved only if it is recorded when the experiment is performed.

Within the FAIR framework [59], the current zirconia ageing literature is largely findable and accessible, since articles are indexed and available, but it is only weakly interoperable and reusable, because experimental parameters are embedded in prose rather than stored as machine-actionable metadata. MIAZE addresses these two principles. Recording each parameter as a defined field, with unreported values entered explicitly as null, allows the completeness and comparability of datasets to be assessed computationally rather than by manual extraction from PDF text. The illustrative baseline in Supplementary Section S4 should be read in this light: it shows the kind of gap that MIAZE is designed to close, not the prevalence of that gap across the literature.

MIAZE follows the minimum-information tradition established by MIAME for microarray experiments [60] and MIAPE for proteomics [61], which define what must be reported for an experiment to be interpreted and reproduced. In dentistry, CRIS [62], PRILE [63], and guidelines for in vitro studies on dental materials [64] address reporting quality, but as human-readable checklists rather than data structures, and at a level too general to specify zirconia processing or ageing parameters. ISO 13356 and ISO 6872 prescribe test methods and conditions, including accelerated ageing in ISO 13356, but not the information that must accompany results for reuse. Materials-science infrastructures such as NOMAD [65] and OPTIMADE [66] provide machine-readable schemas but were developed primarily for computational and crystallographic data, and the Elementary Multiperspective Material Ontology (EMMO) [67] offers a general semantic framework without domain-specific terms for dental restorations. MIAZE is intended to combine the domain content of a reporting checklist with a machine-readable structure; its terms could be mapped to EMMO where equivalents exist.

A MIAZE-compliant dataset consists of a dataset block and four minimum reporting elements, summarised in Table 4: a material baseline (manufacturer, lot, composition, specimen design, processing, and initial state), the experimental increments applied (independent variables such as time, temperature, chemical environment, or load cycles), the response observations directly measured (phase composition, mechanical strength, optical properties, or equivalent outcomes), and the measurement metadata describing how those observations were acquired (instrumentation, acquisition parameters, resolution, calibration, uncertainty). JSON was selected as the initial implementation format because it is open, platform-independent, human-readable, and machine-processable, and because JSON schemas can be validated programmatically with standard validation libraries, so that each dataset can be checked against the schema before it is reused. The full schema definition, validation rules, worked examples, and an illustrative assessment of how completely published studies can be encoded are provided in the Technical Annex (Supplementary Sections S1–S4) and as machine-readable Supplementary Files; this section presents the conceptual architecture only.

**Listing 1.** Minimal MIAZE record (abridged from Supplementary Section S3; one zone, one hydrothermal stage, one response type).
{

 "miaze_version": "0.2.0",

 "dataset": {"identifier": "10.5281/zenodo.0000000", "license": "CC-BY-4.0", ...},

 "material_baseline": {"manufacturer": "Manufacturer X", "lot": "A123",

    "compositional_class": "3Y", "design": "multilayer", "zone_id": "cervical",

    "processing": {"sintering": {"peak_temperature": {"value": 1450, "unit": "Cel"}, ...},

                   "surface_finish": "polished"}, ...},

 "experimental_increments": [{"stage": 1, "stress_type": "hydrothermal",

    "conditions": {"temperature": {"value": 134, "unit": "Cel"},

                   "pressure": {"value": 0.2, "unit": "MPa"}, "medium": "steam"},

    "variable": "time", "unit": "h", "values": [0, 5, 10, 20]}],

 "response_observations": [{"response_type": "monoclinic_fraction", "unit": "%",

    "derivation_method": "Toraya", "stage": 1,

    "points": [{"increment_value": 5, "n": 3, "mean": 8.1,

                "dispersion": {"value": 1.2, "unit": "%", "type": "SD"}}, ...]}],

 "measurement_metadata": [{"response_type": "monoclinic_fraction", "technique": "XRD",

    "uncertainty": {"value": 0.5, "unit": "%", "type": "SD"}, ...}]

}


**Table 5:** Comparison of MIAZE with existing minimum-information standards, reporting checklists, test standards, and materials-science data infrastructures. ✓, covered; ~, partly covered; ×, not covered.

Framework	Domain	Format	Machine-Readable	Zirconia Processing and Ageing Parameters
MIAME [60]	Microarray experiments	Minimum-information standard	~ (via MAGE-ML/MAGE-TAB)	×
MIAPE [61]	Proteomics	Minimum-information standard	~ (via PSI formats)	×
CRIS [62]	Dental in vitro studies	Reporting checklist	×	×
PRILE [63]	Laboratory studies in endodontology	Reporting checklist	×	×
Faggion [64]	In vitro studies on dental materials	Reporting guideline	×	~ (generic items only)
ISO 13356/ISO 6872	Zirconia and dental ceramics	Test standards	×	~ (test conditions only)
NOMAD [65]	Materials science (mainly computational)	Repository and metadata schema	✓	×
OPTIMADE [66]	Crystal-structure databases	API specification	✓	×
EMMO [67]	Materials science	Ontology	✓	×
MIAZE (this work)	Zirconia ageing experiments	Proposed minimum-information framework and JSON schema	✓	✓

MAGE-ML, MicroArray Gene Expression Markup Language; MAGE-TAB, MicroArray Gene Expression Tabular format; PSI, Proteomics Standards Initiative (Human Proteome Organisation) data formats.

### Limitations of MIAZE

MIAZE standardises how experiments are recorded; it does not make an experiment well designed or its measurements accurate. A fully compliant record can still describe an underpowered study, an inappropriate ageing protocol, uncalibrated instruments, or biased specimen selection, and structured formatting may lend such data unwarranted credibility. Compliance should therefore be read as a statement about completeness and reusability, not about quality; users pooling MIAZE datasets still need to apply study-level risk-of-bias appraisal (e.g., the QUIN tool [68]), since reporting checklists such as CRIS [62] assess the completeness of reporting rather than study quality. In addition, MIAZE cannot recover information that was never measured, such as undisclosed manufacturing parameters, and its usefulness depends on community uptake, curation, and version control of the schema, none of which is yet established.

## 5. Downstream Capabilities Enabled by MIAZE

### 5.1. Data Synthesis and Meta-Analysis

The immediate consequence of separating data reporting from data interpretation is that the same MIAZE-compliant dataset can support multiple, independently chosen analytical approaches without requiring the underlying data to be restructured for each one. Table 3 (Section 3) already illustrated this: the methodological limitations that currently fragment the ageing literature (speculative clinical time extrapolation, equipment-specific variability, heterogeneous specimen geometries, and qualitative or pass/fail reporting) map onto a corresponding set of analytical strategies that only become usable once the raw observations are captured completely and consistently.

Rather than relying on fixed analytical paradigms or speculative time-based extrapolation alone, structured, machine-readable datasets are designed to enable several complementary directions. Reference-based normalisation can contextualise material performance against a defined benchmark (e.g., 3Y-TZP) to reduce inter-laboratory variability, provided the reference choice is reported explicitly and independently of the data format itself. Methodological harmonisation, such as controlled specimen geometries or parallel testing across hydrothermal, mechanical, and chemical stressors, improves comparability without forcing every laboratory onto a single protocol. And quantitative reporting of degradation behaviour, using continuous response variables rather than qualitative pass/fail outcomes, allows the same dataset to be interpreted through classical kinetic modelling, statistical regression, or data-driven machine-learning techniques, depending on the research question. For example, Mehl–Avrami–Johnson kinetics [57] can be fitted to monoclinic-fraction series, and Weibull statistics [58] to per-specimen strength values, both from the same record. None of these approaches requires modification to the underlying MIAZE structure; they are simply different lenses applied above the reporting layer.

MIAZE can help resolve the time–temperature conversion problem, but only under specific preconditions. First, datasets must contain isothermal series at three or more temperatures for the same material and finish, so that an apparent activation energy can be estimated rather than assumed. Second, low-temperature (e.g., 37 to 70 °C) or real-time data, and ideally clinically retrieved restorations with known service time, are needed as anchors, because extrapolation from 134 °C alone cannot validate itself. Third, the monoclinic fraction must be derived with a stated method and comparable probe depth. If these conditions are met, pooled MIAZE datasets would allow conversion factors to be recalculated per material class rather than borrowed from orthopaedic 3Y-TZP; if they are not met, MIAZE only makes the existing uncertainty transparent.

These benefits apply to data recorded prospectively in MIAZE format. Published studies, including our own, can be mapped onto the MIAZE structure, but not completely (Supplementary Section S4).

This is also the basis on which MIAZE datasets become viable inputs for machine-learning applications specifically: consistent, complete, per-experiment records of increments and response observations are precisely the structured input format that model training and testing require, without MIAZE itself dictating which model is appropriate. As harmonised datasets accumulate across laboratories, the same infrastructure that enables manual meta-analysis today extends naturally to larger-scale, computational data mining tomorrow.

### 5.2. Conceptual Material-Selection Scheme (Illustrative)

**Figure 6:**
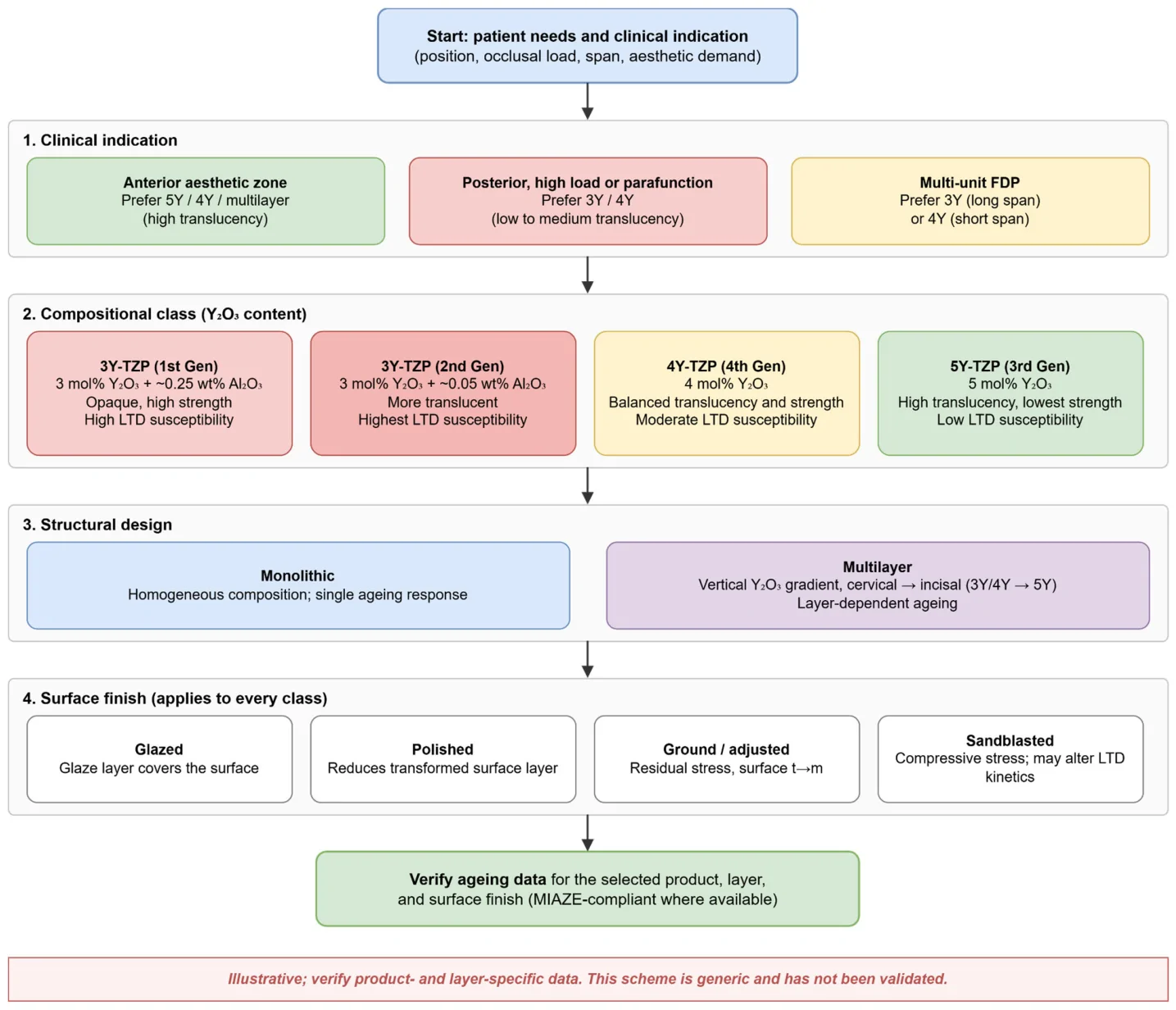
Conceptual material-selection scheme for zirconia fixed restorations, proceeding from clinical indication to compositional class, structural design, and surface finish. The scheme summarises published evidence for each step: (1) clinical indication [5,6,17,20,32]; (2) compositional class and relative LTD susceptibility [17,20,21,22,25,26]; (3) structural design and layer-dependent ageing [32,33,34]; (4) surface finish [23,24,36,69]; it is illustrative, generic, and has not been validated, and ageing data should be verified for the specific product, layer, and surface finish.

The preceding sections establish MIAZE as data infrastructure; this section illustrates why that infrastructure matters clinically and not only analytically. Zirconia’s compositional generations, structural designs, and ageing behaviour, surveyed in Section 2, are individually well characterised. What clinicians actually need, however, is a unified interpretation of how composition, microstructure, and ageing behaviour collectively determine material suitability for a specific patient and a specific restoration, since patient needs are governed in large part by the fundamental trade-off between aesthetic translucency and mechanical performance that yttria content controls (Figure 6).

Figure 6 consolidates these considerations into a conceptual scheme that proceeds from the clinical indication (anatomical position, occlusal load, span, and aesthetic demand) through compositional class and structural design to surface finish, which modifies LTD susceptibility independently of composition [23,24,69]. Because the scheme operates at the level of compositional class, it shares the limitation shown in Figure 2: ageing data must be verified for the specific product, layer, and surface finish. Its reliability therefore depends on comparable input data of the kind MIAZE is designed to preserve.

Looking forward, it is worth being precise about what MIAZE does and does not claim. MIAZE is a data-delivery layer, not a clinical tool, an analytical model, or a machine-learning system. Its role is analogous to that of standardised tabular and array formats (e.g., CSV, Parquet, or NumPy arrays), which libraries such as pandas and scikit-learn can read directly. Such formats do not themselves classify, predict, or diagnose anything; they guarantee that data produced by one group can be used without reformatting by any downstream tool a different group chooses to build, whether that tool is a plotting script, a kinetic-model fitter, a statistical test, or a machine-learning classifier. MIAZE aims to occupy the same position for zirconia ageing data: by fixing the structure of material_baseline, experimental_increments, response_observations, and measurement_metadata, it gives future independent researchers and software developers a stable, predictable input format to build against.

Whether that eventually includes clinical decision-support software that cross-references patient-specific risk factors (e.g., bruxism, acidic dietary exposure, oral hygiene) against harmonised material performance profiles is, at this stage, an open question for the field; it is not a capability that this paper proposes, demonstrates, or claims to deliver. No data-driven decision-support software of this kind exists today; the conceptual scheme in Figure 6 is a static summary of published evidence, not such a tool. Building one is explicitly outside MIAZE’s scope; MIAZE’s only claim is that, if such a tool is built, standardised data will be available for it to consume.

In summary, clinical material selection is best served by an integrated workflow rather than by a focus on isolated material properties. The scheme in Figure 6 illustrates that zirconia performance is a dynamic outcome of restoration design, compositional class, and site-specific functional demand, and that reconciling the trade-off between aesthetic translucency and mechanical reliability depends on the same standardised, comparable data that Section 4 and Section 5.1 establish as the paper’s central proposal.

## 6. Conclusions and Future Work

Artificial ageing of zirconia-based materials in fixed prosthodontics currently relies on a fragmented spectrum of methodologies, each constrained by mechanistic limitations and uncertain clinical correlations. The widespread reliance on hydrothermal ageing as the principal evaluation method for 3Y-TZP is increasingly inadequate for newer translucent 4Y-TZP and 5Y-TZP generations, which exhibit substantially different degradation behaviour, and the unresolved discrepancy between competing time–temperature equivalency factors illustrates the limits of estimating clinical longevity through temporal extrapolation alone.

As established throughout this review, however, the central obstacle is not primarily a shortage of ageing protocols; the field already has more protocols than it can compare. Rather than relying on disputed service-year conversions, future studies would benefit from emphasising experimentally observed degradation behaviour, supported by transparent and reproducible reporting of experimental conditions and measurements. Standardised reporting through the MIAZE framework provides the model-neutral, machine-readable structure that makes this possible: by defining the structure, completeness, and interoperability of reported observations without prescribing the analytical methods used for subsequent interpretation, MIAZE is intended to address the reporting/interpretation conflation diagnosed in Section 3, not by proposing a competing protocol but by removing the need to agree on one before data can be meaningfully compared.

As zirconia materials and analytical technologies continue to evolve, preservation of structured experimental information may become as important as the development of new testing methodologies themselves. Once standardised datasets are available, multiple analytical approaches, such as kinetic modelling, statistical regression, probabilistic analysis, or data-driven computational methods, may be applied according to the research objective and dataset structure, as outlined in Section 5. No single analytical approach is universally required; analytical selection should remain dependent on the scientific question, the characteristics of the dataset, and the assumptions of the selected model. Other ageing modalities, namely thermocycling, chewing simulation, and chemical immersion, remain valuable complementary approaches but continue to suffer from substantial methodological heterogeneity; greater harmonisation of protocols, specimen characterisation, and outcome reporting would substantially improve inter-study comparability and future evidence synthesis.

Future research should therefore prioritise: generation-specific ageing protocols that reflect the differing susceptibilities of 3Y-TZP, 4Y-TZP, 5Y-TZP, and multilayer zirconia systems; comprehensive, machine-readable reporting of experimental observations and metadata through standardised frameworks such as MIAZE; transparent reporting of sufficient raw experimental data to enable independent post hoc analyses using diverse analytical methodologies; continued investigation of normalisation strategies, including reference materials such as high-performance 3Y-TZP, to improve cross-study comparability where appropriate; long-term validation through clinical retrieval studies capable of linking laboratory observations with real-world material performance; and, once studies report in MIAZE format, automated assessment of reporting completeness across the literature, replacing manual extraction from PDF text. The same four MIAZE blocks can document retrieved restorations: material_baseline (manufacturer, lot where known, compositional class, zone, surface finish at delivery), experimental_increments replaced by a clinical exposure record (stress type “clinical_service”, time in situ, tooth position, antagonist, bruxism, reason for removal), response_observations (regional monoclinic fraction, roughness, fractographic findings), and unchanged measurement_metadata, allowing retrieved and laboratory-aged specimens to be compared in one data structure. Generation-specific protocols remain desirable, but they will inevitably lag behind new materials; a common reporting format ensures that data obtained with current protocols, and with any future generation-specific protocol, remain comparable.

Until dedicated, generation-specific standards become available, researchers are encouraged to employ sufficiently demanding ageing protocols, including extended hydrothermal exposure where scientifically justified and complementary mechanical and environmental challenges, while placing emphasis on transparent reporting of experimentally observed material behaviour rather than uncertain predictions of clinical service life based solely on disputed temporal equivalency factors. Such an approach provides a robust foundation for reproducible research, future meta-analysis, and the continued development of analytical strategies for understanding zirconia ageing across evolving material generations. The central challenge in zirconia ageing research is therefore not merely the development of additional protocols, but the preservation of experimental information in a structure that allows meaningful comparison across evolving materials, methodologies, and future analytical approaches. Providing this structural prerequisite is the purpose of MIAZE.

## Figures and Tables

**Table 1:** Comparative summary of the principal artificial ageing methodologies applied to zirconia in fixed prosthodontics, giving the mechanism assessed, representative parameter ranges reported in the literature, typical instrumentation and outcome measures, the principal limitations of each approach, and the closest applicable ISO or ASTM reference.

Method	Parameters Reported in the Literature	Equipment	Outcomes Assessed	Limitations	ISO/ASTM Ref.
Hydrothermal autoclave (hydrothermal domain)	110–140 °C; 0.2–2 bar; 1–64 h. Most common: 134 °C, 0.2 MPa (2 bar), 5 h (ISO 13356).	Autoclave (steam-pressure vessel)	Phase transformation (XRD), flexural strength, surface roughness, SEM, microhardness	Highly accelerated; time–temperature extrapolation debated; conversion factors calibrated on 3Y-TZP are not transferable to 4Y/5Y-TZP	ISO 13356:2015 [9] (orthopaedic implant grade; benchmark only)
Thermocycling (thermomechanical domain)	5–55 °C (most common); 5–60 °C or 5–75 °C for greater stress. Cycles: 600–50,000 (10,000 ≈ 1 year). Dwell: typically 20–30 s (range 3–60 s).	Thermocycler/water-bath device	Bond strength, microhardness, colour (ΔE), marginal integrity, phase content	Wide variability in cycle number, dwell time and temperature range; poor inter-study comparability	ISO/TS 11405 [38] (adhesion testing only)
Chewing/masticatory simulation (thermomechanical domain)	1.2 × 10^6^ cycles (≈5 years); 50–200 N; 1.0–1.6 Hz; 37 °C water bath. Often combined with thermocycling.	Chewing simulator (e.g., Willytec/SD Mechatronik; Regensburg type)	Fracture load, survival, surface wear, phase transformation, fatigue degradation	Variable loads and cycles; no single accepted protocol; expensive equipment required	None specific (ISO 14801 [39] applies to implant fatigue only)
Chemical immersion (chemical domain)	4% acetic acid at 60–80 °C; HCl pH 1.2; artificial saliva at 37 °C; NaOH; varies widely.	Temperature-controlled bath/oven	Phase transformation, elemental release (ICP), surface roughness, colour stability	Poorly standardised; acid kinetics differ from hydrothermal ageing; limited clinical correlation	ISO 6872 [40] (chemical solubility test)
UV/photoageing (optical domain)	340 nm, 0.55 W/m^2^; 300–1500 h (Weather-Ometer or UV chamber). Also 365 nm.	UV ageing chamber, Weather-Ometer	Colour stability (CIE ΔE, ΔE_00_), surface roughness, translucency parameter, microhardness	Primarily relevant for extrinsic colouring; zirconia crystalline structure is UV-stable; low structural relevance	ASTM G154 [41]; ISO 7491 [42] (colour stability)
Clinically related/combined	Chewing simulation (1.2 × 10^6^ cycles) + thermocycling (5000–50,000 cycles) + chemical immersion (HCl, pH 1.2, 15 h). Sequence varies.	Multiple devices (simulator + thermocycler + bath)	Fracture resistance, optical changes, translucency, colour, surface condition	No protocol consensus; heterogeneous combinations; high resource demand; difficult to isolate variable effects	None defined

ISO, International Organization for Standardization; ASTM, ASTM International (formerly American Society for Testing and Materials); ISO/TS, ISO Technical Specification, a document published by ISO before consensus for a full International Standard is reached; XRD, X-ray diffraction; SEM, scanning electron microscopy; ICP, inductively coupled plasma; ΔE, colour difference.

**Table 2:** Illustrative, non-exhaustive post hoc analytical representations, organised as a decision matrix linking a research objective to a material system, an experimental stressor, and an example (non-required) analytical interpretation. The matrix presupposes laboratory data captured at MIAZE-compliant resolution.

Research Objective	Material System	Experimental Stressor	Example Analytical Interpretation (Non-Required)
Material stability comparison	3Y, 4Y, 5Y (monolithic zirconia)	Autoclave ageing time (hydrothermal exposure)	Phase-transformation kinetic descriptor (example kinetic descriptor)
Fatigue resistance mapping	Multilayer zirconia systems	Mechanical loading (cycles/force amplitude)	Mechanical degradation rate (example degradation metric)
Chemical/corrosion response	3Y, 4Y, 5Y (flat disc specimens)	pH exposure/chemical immersion duration	Surface degradation index (example response slope, model-dependent)
Multi-variable optimisation	All zirconia variants	Combined stressors (temperature, pH, load, time)	Multidimensional stability vector (feature-space representation)

**Table 3:** Recurring methodological limitations in the zirconia ageing literature mapped onto representative post hoc analytical approaches, with the rationale linking each approach to the specific MIAZE reporting element that makes it applicable. The mapping is interpretive rather than prescriptive.

Current Narrative Limitation	Example Analytical Approaches	Rationale
Speculative clinical time extrapolations	Kinetic modelling, statistical regression	Relies on raw experimental increments and response observations rather than assumed clinical-year equivalencies, so time-extrapolation claims can be recalculated as models improve, provided that isothermal data at several temperatures, or real-time data at body temperature, are captured.
Equipment-specific variability	Reference normalisation, statistical calibration	Uses measurement metadata (instrumentation, calibration, uncertainty) to correct for inter-laboratory differences instead of treating results as directly comparable.
Heterogeneous specimen geometries	Geometry-stratified subgroup analysis; normalisation by exposed surface area	Captures specimen geometry and surface finish within the material baseline so that their effects can be analysed separately.
Qualitative or pass/fail reporting	Continuous response modelling, probabilistic methods	Requires response observations to be reported as measured values rather than binary outcomes, preserving the granularity needed for quantitative modelling.

**Table 4:** The MIAZE reporting schema. The dataset block and the four minimum reporting elements are listed with their required JSON keys, required observational fields, and the rationale by which each element preserves observation primacy at the data-infrastructure layer.

Data Category	Required JSON Key	Required Observational Fields	Rationale for Observation Primacy
Dataset	dataset	Persistent identifier, licence, creators (ORCID), date, encoding type, source publication, raw-data link	Establishes FAIR provenance and distinguishes original from re-encoded data
Material baseline	material_baseline	Manufacturer, product, lot, compositional class, yttria (mol%), alumina (wt%), shade, design, zone_id (multilayer), processing (sintering temperature, dwell, heating/cooling rates; surface finish), specimen geometry, initial state (phase fractions, grain size, density, optional lattice parameters)	Establishes the immutable provenance and composition of the specimen before experimental stress
Experimental increments	experimental_increments	Ordered stages; per stage: stress type, conditions required by stress type (e.g., temperature, pressure, medium for hydrothermal ageing), independent variable, unit, values	Documents the raw stress conditions applied, without analytical transformation or temporal extrapolation
Response observations	response_observations	Response type, unit, derivation method, zone_id, stage; per point either per-specimen values or mean, n, and dispersion	Records direct experimental outcomes; excludes derived kinetic slopes, regression coefficients, or fitted parameters
Measurement metadata	measurement_metadata	Per response type: technique, instrument, acquisition parameters, calibration, numeric uncertainty (value, unit, type)	Ensures technical reproducibility by documenting the precision and conditions of data acquisition

In practical terms, a MIAZE record works like a structured laboratory form with fixed, named fields: each field holds one piece of information (for example, sintering temperature with its unit), and a field that was not measured is marked as not reported rather than left out. JSON is simply the file format in which this form is stored, so that it can be read both by researchers and by software; no programming is needed to fill it in, and the provided validation script checks a completed record automatically. A minimal example record is shown in Listing 1. Table 5 compares MIAZE with the existing reporting frameworks and data infrastructures discussed above.

## Data Availability

No new experimental datasets were generated in this study. The MIAZE version 0.2.0 schema, validation and plotting scripts, example records, and the bibliometric script underlying Figure 4 are provided in the Supplementary Materials and archived at https://doi.org/10.5281/zenodo.23010480.

## Supplementary Materials

The following supporting information can be downloaded at: https://www.mdpi.com/article/10.3390/jfb17100515/s1. Technical Annex (Sections S1–S4: MIAZE version 0.2.0 schema description, validation demonstration, worked example, and illustrative assessment of published studies); Supplementary Section S5 (bibliometric search strategy and annual counts, Table S2); archive MIAZE_Schema_and_Python_Tools.zip containing schema/miaze_schema.json, scripts/validate_miaze.py, scripts/plot_miaze.py, scripts/pubmed_bibliometrics.py, data/example_original.json, data/example_legacy_reencoded.json, and data/example_invalid.json.
