# Supplementary material for: Artificial Ageing Methodologies for Zirconia-Based Restorations in Fixed Prosthodontics: A Narrative Review and Proposal of the MIAZE Reporting Framework
Source: J Funct Biomater. 2026 Oct 13;17(10):515. doi: 10.3390/jfb17100515 (PMC13706288; doi:10.3390/jfb17100515)
Supplement: Supplementary file 1 [file jfb-17-00515-s001.zip › jfb-4583262-supplementary.pdf]

Journal of
Functional Biomaterials

MDPI

Supplementary Materials

Artificial Ageing Methodologies for Zirconia-Based Restorations in Fixed Prosthodontics: A Narrative Review and Proposal of the MIAZE Reporting Framework

Marta Banjšak, Mark Žic and Marko Jakovac

Contents: Technical Annex, Sections S1 to S4 (MIAZE version 0.2.0 schema description, validation demonstration, worked example, and illustrative assessment of published studies); Section S5 (bibliometric search strategy and annual counts, Table S2). Machine-readable files (schema, scripts, and example records) are provided in the accompanying archive MIAZE_Schema_and_Python_Tools.zip and archived at https://doi.org/10.5281/zenodo.23010480.

Technical Annex: Sections S1 to S4

Minimum Information About a Zirconia Experiment (MIAZE), version 0.2.0

Section S1: MIAZE Formal Schema

S1.1 Scope

MIAZE defines a minimal, model-neutral reporting framework for zirconia-based experimental studies in fixed prosthodontics. It is designed to establish the essential information required to preserve scientific interpretability, reproducibility, and future reuse of experimental datasets. Importantly, MIAZE does not require researchers to adopt identical experimental protocols, analytical methods, or instrumentation. Instead, it provides a standardised structure for documenting experimental observations and associated metadata, allowing heterogeneous studies to remain comparable while preserving the flexibility required for different research objectives.

MIAZE specifies data reporting only and does not prescribe analytical frameworks, mathematical models, or interpretation strategies. It also does not guarantee experimental quality: a compliant record can describe a poorly designed or poorly executed study (see the subsection Limitations of MIAZE in Section 4 of the main text).

MIAZE is a prospective proposal. It defines how future studies should record their data and is not intended as a tool for re-encoding the existing literature. Its role is comparable to that of a JSON Schema, which specifies the structure of documents created against it: documents written before the schema existed can often be read, but rarely conform to it.

S1.2 Core Reporting Principles

- **Observation primacy**: measured quantities are reported, including conventionally derived ones (e.g., monoclinic fraction, flexural strength, colour difference ΔE_{00}), provided the derivation method is stated. Model-fitted parameters (rate constants, activation energies, Weibull moduli) are excluded from the minimum layer. Raw data (diffractograms, load–displacement curves) should be deposited and linked.

- **Model neutrality**: the structure is independent of any specific kinetic, statistical, or machine-learning model.
- **Interoperability**: data shall be encoded in structured key–value format (JSON or equivalent) using controlled vocabularies, UCUM unit codes (Unified Code for Units of Measure, a standard set of machine-readable unit symbols such as Cel, MPa, h, and %), and numeric uncertainty objects.
- **Complete metadata context**: experimental conditions, instrumentation, specimen provenance, and processing history must be documented.
- **Explicit non-reporting**: every required key must be present. Required keys are defined conditionally, so they always apply to the record in question; for example, pressure is required only for hydrothermal stages and zone_id only for multilayer materials. A value that was not measured or not reported is recorded as null, never omitted, so that missing information remains visible and measurable (Section S1.6). An optional key that does not apply to a study is omitted.

The principles underlying MIAZE align with the FAIR data principles [59] by promoting findability, accessibility, interoperability, and reuse of experimental information. Reference numbers in this document refer to the reference list of the main article.

S1.3 Minimum Reporting Elements

A MIAZE-compliant dataset SHALL contain the following components (full definition: schema/miaze_schema.json in the accompanying archive):

- **Dataset** (dataset): persistent identifier (DOI), licence (SPDX), creators with ORCID iDs, date, encoding type (original or legacy re-encoded), source publication, and link to raw data.
- **Material baseline** (material_baseline): manufacturer, product, lot (batch) number, compositional class, yttria content (mol%), alumina content (wt%), shade, specimen design, layer/zone identifier (zone_id; required for multilayer materials); processing (sintering peak temperature, dwell, heating and cooling rates, furnace, program; surface finish); specimen geometry and dimensions; and initial state (monoclinic and cubic fractions, grain size, relative density, optional tetragonal lattice parameters a and c).
- **Experimental increments** (experimental_increments): an ordered array of stages. Each stage records its stress type, the independent variable and its values, and a conditions object whose required keys depend on the stress type: hydrothermal (temperature, pressure, medium); thermocycling (lower and upper temperature, dwell, medium); cyclic mechanical loading (load, frequency, antagonist, medium); chemical (medium, pH, temperature). Sequential and combined protocols are encoded as several stages.
- **Response observations** (response_observations): one entry per response type, each with unit, derivation method, zone_id, and the stage it refers to. Each data point contains either per-specimen values with specimen identifiers (required for destructive endpoints such as flexural strength and fracture load, enabling Weibull analysis) or the mean, n, and a dispersion object. Several response types share one record.
- **Measurement metadata** (measurement_metadata): one entry per response type: instrumentation, acquisition parameters, calibration method, and associated uncertainty as a numeric object (value, unit, type: SD, SE, CI95, expanded, range, or instrument resolution; coverage factor k).

S1.4 Data Format Specification (JSON)

JSON was selected as the initial implementation format for MIAZE because it provides an open, platform-independent, human-readable, and machine-processable structure for representing experimental observations and associated metadata. Study-specific

attributes may be added only inside a dedicated extensions object, using keys with the prefix x_. Extensions must not redefine or duplicate core fields, are ignored when compliance is assessed, and should reference a public definition; keys outside the defined structure are rejected by the validator. This keeps records extensible without eroding interoperability. The schema is enforced with standard validation libraries (e.g., Python's jsonschema), as demonstrated in Section S2. A complete worked example is provided in Section S3.

S1.5 Analytical Layer Separation

MIAZE constitutes a data infrastructure layer only. Analytical interpretation is performed post hoc using independently defined methodologies, including classical kinetic models, statistical regression, probabilistic methods, and data-driven machine-learning approaches. Separation of data acquisition from analytical interpretation ensures long-term reusability of datasets independent of evolving modelling paradigms.

S1.6 Compliance, Completeness, and Legacy Data

MIAZE distinguishes two properties of a record. Compliance means that the record has the required structure and every required key (checked by the schema). Completeness is the percentage of required and recommended fields holding a reported value rather than null. New studies are expected to reach full completeness. Published (legacy) studies can usually be encoded in a compliant structure, but many fields must be set to null because the original articles do not report them, for example measurement uncertainty, per-specimen values, or disc lot numbers. MIAZE cannot recover such information retrospectively; it makes the gap explicit and quantifiable. This historical under-reporting is the primary justification for MIAZE: no common reporting structure has existed for zirconia ageing studies, and the missing information can be preserved only if it is recorded when the experiment is performed. MIAZE is therefore intended for adoption in future studies; legacy re-encoding serves only to document the extent of the gap (Section S4).

Section S2: Practical Demonstration of MIAZE Implementation

MIAZE-compliant datasets are intended for deposition in machine-readable repositories and integration into computational analysis pipelines. The schema, validator, and examples (archive MIAZE_Schema_and_Python_Tools.zip) are archived in a permanent public repository [https://doi.org/10.5281/zenodo.23010480].

S2.1 JSON Schema Validation

The Python function validate_miaze (scripts/validate_miaze.py) validates datasets against the formally defined schema and reports both compliance and completeness, listing every field recorded as not reported. Three example files demonstrate its behaviour: data/example_original.json (a fully reported record: compliant, 100% complete); data/example_legacy_reencoded.json (a constructed record that imitates the reporting typical of published studies and is not a re-encoding of any specific study: compliant, 57.9% complete, with 16 fields not reported, including sintering heating and cooling rates, surface finish, and autoclave pressure); and data/example_invalid.json, which is rejected because the autoclave pressure key is missing, the uncertainty is given as the free-text string "±0.5%", and an extension key lacks the x_ prefix.

S2.2 Data Plotting: Illustrating Computational Reuse

Loading a MIAZE-compliant file, the plotting script (scripts/plot_miaze.py) directly extracts experimental_increments (x-axis) and response_observations (y-axis) and generates a plot without manual preprocessing, illustrating that the choice of analytical tool remains independent of the data structure.

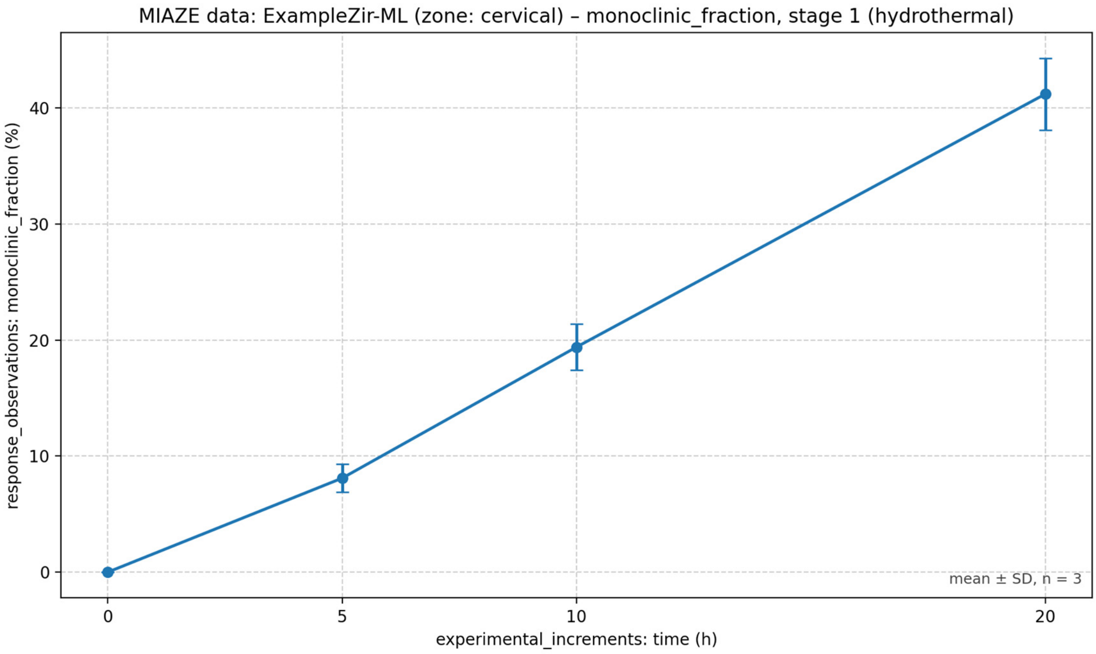

Figure S1. Direct visualisation of a MIAZE-compliant dataset, generated without prior data manipulation. Monoclinic phase fraction (%; mean ± SD, n = 3) is plotted against hydrothermal ageing duration (h) for the illustrative dummy record in Section S3 (data/example_original.json). The data are illustrative and not experimental.

Section S3: Sample MIAZE-Compliant JSON Payload

The following record (data/example_original.json) is an illustrative, dummy dataset for a multilayer 3Y-TZP cervical zone with two response types. It is not experimental data.

```
{
  "miaze_version": "0.2.0",
  "dataset": {
    "identifier": "10.5281/zenodo.0000000",
    "encoding_type": "original",
    "license": "CC-BY-4.0",
    "creators": [{"name": "Example Author", "orcid": null}],
    "date": "2026-09-23",
    "raw_data_uri": null
  },
  "material_baseline": {
    "manufacturer": "Manufacturer X",
    "product": "ExampleZir-ML",
    "lot": "A123",
    "compositional_class": "3Y",
    "yttria_mol_pct": 3.0,
    "alumina_wt_pct": 0.05,
    "shade": "A2",
    "design": "multilayer",
    "zone_id": "cervical",
    "processing": {
      "sintering": {
        "peak_temperature": {"value": 1450, "unit": "Cel"},
        "dwell": {"value": 120, "unit": "min"},
        "heating_rate": {"value": 10, "unit": "Cel/min"},
        "cooling_rate": {"value": 10, "unit": "Cel/min"},
        "furnace": "Furnace Y",
        "program": "conventional"
      },
      "surface_finish": "polished",
      "surface_finish_details": "diamond paste to 1 um"
    },
    "specimen": {
      "geometry": "disc",
      "dimensions": [{"value": 12.0, "unit": "mm"}, {"value": 1.2, "unit": "mm"}]
    },
    "initial_state": {
      "monoclinic_fraction": {"value": 0.0, "unit": "%"},
      "cubic_fraction": {"value": 14.0, "unit": "%"},
      "grain_size": {"value": 0.45, "unit": "um"},
      "relative_density": {"value": 99.5, "unit": "%"},
      "tetragonal_lattice": {
        "a": {"value": 3.604, "unit": "Ao"},
        "c": {"value": 5.176, "unit": "Ao"}
      }
    }
  },
  "experimental_increments": [
    {
      "stage": 1,
      "stress_type": "hydrothermal",
      "variable": "time",
      "unit": "h",
      "values": [0, 5, 10, 20],
      "conditions": {
        "temperature": {"value": 134, "unit": "Cel"},
        "pressure": {"value": 0.2, "unit": "MPa"},
        "medium": "steam"
      }
    }
  ],
  "response_observations": [
    {
      "response_type": "monoclinic_fraction",
      "unit": "%",
      "derivation_method": "Toraya",
      "zone_id": "cervical",
      "stage": 1,
      "points": [
        {
          "increment_value": 0,
          "n": 3,
          "mean": 0.0,
          "dispersion": {"value": 0.0, "unit": "%", "type": "SD"}
        },
        {
          "increment_value": 5,
```

```
          "n": 3,
          "mean": 8.1,
          "dispersion": {"value": 1.2, "unit": "%", "type": "SD"}
        },
        {
          "increment_value": 10,
          "n": 3,
          "mean": 19.4,
          "dispersion": {"value": 2.0, "unit": "%", "type": "SD"}
        },
        {
          "increment_value": 20,
          "n": 3,
          "mean": 41.2,
          "dispersion": {"value": 3.1, "unit": "%", "type": "SD"}
        }
      ]
    },
    {
      "response_type": "flexural_strength",
      "unit": "MPa",
      "derivation_method": "ISO 6872 biaxial (piston-on-three-ball)",
      "zone_id": "cervical",
      "stage": 1,
      "points": [
        {
          "increment_value": 0,
          "specimen_values": [
            {"specimen_id": "S01", "value": 1012},
            {"specimen_id": "S02", "value": 968}
          ]
        },
        {
          "increment_value": 20,
          "specimen_values": [
            {"specimen_id": "S11", "value": 941},
            {"specimen_id": "S12", "value": 903}
          ]
        }
      ]
    }
  ],
  "measurement_metadata": [
    {
      "response_type": "monoclinic_fraction",
      "technique": "XRD (Bragg-Brentano)",
      "instrument": "Diffractometer Z",
      "acquisition_parameters": {
        "radiation": "Cu Ka",
        "two_theta_range_deg": [27, 33],
        "step_deg": 0.02
      },
      "calibration": "Si standard",
      "uncertainty": {"value": 0.5, "unit": "%", "type": "SD", "coverage_factor_k": null}
    },
    {
      "response_type": "flexural_strength",
      "technique": "biaxial flexure",
      "instrument": "Universal testing machine W",
      "acquisition_parameters": {"crosshead_speed_mm_min": 1.0},
      "calibration": "load cell calibrated 2026",
      "uncertainty": {
        "value": 1.0,
        "unit": "%",
        "type": "instrument_resolution",
        "coverage_factor_k": null
      }
    }
  ],
  "extensions": {"x_raman_map_uri": null}
}
```


Section S4: Legacy Data and Reporting Gaps

This appendix provides an illustrative baseline, not a prevalence estimate. Five zirconia studies, four of them by the present authors, were checked against the MIAZE fields

(Table S1). None of the four studies by the present authors involved artificial ageing, so the ageing-specific fields (hydrothermal pressure and medium) could be assessed only in the external study [25]. Each study could be mapped onto the MIAZE structure, but none could be encoded completely: measurement uncertainty was reported in none of the five studies, dispersion (n and SD) was reported in three, and the disc lot number was stated in one. Per-specimen values apply only to the two studies with destructive endpoints [25,35], and neither reported them. These omissions are consistent with publishing conventions that have not required such parameters, rather than with practice specific to individual authors.

A coded audit of a larger sample was not undertaken. Because deciding whether a parameter is absent or only ambiguously reported is partly subjective, and manual extraction is prone to error [55], such an audit would require dual independent coding of a defined set of hydrothermal-ageing studies with an inter-coder agreement statistic. In its absence, statements about reporting gaps in the main text are restricted to the five studies in Table S1, and the prevalence of these gaps across the literature remains to be established. Once studies report in MIAZE format, reporting completeness can be computed automatically (Section S2.1). Where a parameter was never reported, it cannot be recovered by any method; this is the practical justification for adopting MIAZE prospectively.

Table S1. Illustrative reporting of selected MIAZE fields in five published studies, including four by the present authors (✓, reported; ×, not reported; ~, reported ambiguously; n/a, not applicable because no hydrothermal ageing or no destructive test was performed).

MIAZE field	Ref. [23]	Ref. [31]	Ref. [35]	Ref. [36]	External [25]
material_baseline.lot	×	×	✓	×	×
processing.sintering.peak_temperature / dwell	×	~	✓	~	✓
processing.sintering.heating_rate / cooling_rate	×	~	✓	×	~
processing.surface_finish	✓	✓	✓	✓	✓
initial_state.grain_size / relative_density	~	~	~	~	×
conditions.pressure (hydrothermal)	n/a	n/a	n/a	n/a	✓
conditions.medium (steam / water / saliva)	n/a	n/a	n/a	n/a	✓
response_observations.derivation_method	×	✓	✓	✓	✓
points: n and dispersion (e.g., SD)	×	×	✓	✓	✓
points: per-specimen values (destructive endpoints)	n/a	n/a	×	n/a	×
measurement_metadata.acquisition_parameters	✓	✓	✓	✓	✓
measurement_metadata.uncertainty	×	×	×	×	×

Section S5: Bibliometric Analysis (Figure 4)

PubMed was queried through the NCBI Entrez E-utilities (esearch) on 23 September 2026. Counts were retrieved per publication year (publication date field [pdat]) without a retrieval cap, using title/abstract [tiab] field restrictions. The four themes were counted independently and may overlap. The number of ageing studies naming an ISO standard was obtained by combining the ageing and ISO queries with AND. Values for 2026 are partial. The analysis was performed with the Python script scripts/pubmed_bibliometrics.py provided in the accompanying archive. Search strings:

Material generations:

```
("3Y-TZP"[tiab] OR "4Y-TZP"[tiab] OR "5Y-TZP"[tiab] OR 3Y[tiab] OR 4Y[tiab] OR 5Y[tiab]
OR "translucent zirconia"[tiab] OR "high-translucency"[tiab] OR multilayer*[tiab] OR
multi-layer*[tiab]) AND (zirconia[tiab] OR "zirconium oxide"[tiab] OR Y-TZP[tiab] OR "yt-
tria-stabilized"[tiab] OR "yttria-stabilised"[tiab]) AND (dental[tiab] OR dentistry[tiab]
```

```
OR prosthodont*[tiab] OR crown*[tiab] OR "fixed dental prosthes*"[tiab] OR restora-
tion*[tiab])
```

Artificial ageing:

```
(aging[tiab] OR ageing[tiab] OR aged[tiab] OR "low-temperature degradation"[tiab] OR hy-
drothermal[tiab] OR autoclav*[tiab] OR thermocycl*[tiab] OR "thermal cycling"[tiab] OR
"chewing simulat*"[tiab] OR "mastication simulat*"[tiab]) AND (zirconia[tiab] OR "zirco-
nium oxide"[tiab] OR Y-TZP[tiab] OR "yttria-stabilized"[tiab] OR "yttria-stabi-
lised"[tiab]) AND (dental[tiab] OR dentistry[tiab] OR prosthodont*[tiab] OR crown*[tiab]
OR "fixed dental prosthes*"[tiab] OR restoration*[tiab])
```

Clinical studies:

```
("clinical trial"[pt] OR "randomized controlled trial"[pt] OR "clinical study"[tiab] OR
"clinical evaluation"[tiab] OR prospective[tiab] OR retrospective[tiab] OR "in
vivo"[tiab] OR patients[tiab] OR retriev*[tiab]) AND (zirconia[tiab] OR "zirconium ox-
ide"[tiab] OR Y-TZP[tiab] OR "yttria-stabilized"[tiab] OR "yttria-stabilised"[tiab]) AND
(dental[tiab] OR dentistry[tiab] OR prosthodont*[tiab] OR crown*[tiab] OR "fixed dental
prosthes*"[tiab] OR restoration*[tiab]) NOT ("in vitro"[tiab] OR "chewing simulat*"[tiab]
OR thermocycl*[tiab])
```

ISO standards:

```
("ISO 13356"[tiab] OR "ISO 6872"[tiab] OR "ISO/TS 11405"[tiab] OR "ISO 14801"[tiab] OR
"ISO standard"[tiab] OR "International Organization for Standardization"[tiab]) AND (zir-
conia[tiab] OR "zirconium oxide"[tiab] OR Y-TZP[tiab] OR "yttria-stabilized"[tiab] OR
"yttria-stabilised"[tiab])
```

Table S2. Annual number of PubMed records per theme, 2016 to 2026. *2026 is a partial year (data retrieved on 23 September 2026).

Year	Material generations	Artificial ageing	Clinical studies	ISO standards	Ageing studies naming ISO	% of ageing studies
2016	27	65	86	11	3	4.6
2017	26	66	92	11	3	4.5
2018	38	95	103	19	3	3.2
2019	50	91	82	9	1	1.1
2020	85	117	96	16	2	1.7
2021	95	146	132	25	5	3.4
2022	114	153	136	19	5	3.3
2023	139	127	134	15	2	1.6
2024	136	125	142	15	3	2.4
2025	145	168	178	16	5	3.0
2026*	124	126	149	18	3	2.4
